# Supplementary material for: Bovine Respiratory Disease: Conventional to Culture-Independent Approaches to Studying Antimicrobial Resistance in North America
Source: Antibiotics (Basel). 2022 Apr 5;11(4):487. doi: 10.3390/antibiotics11040487 (PMC9025279; doi:10.3390/antibiotics11040487)
Supplement: Supplementary file 1 [file antibiotics-11-00487-s001.zip › antibiotics-1649571-supplementary.pdf]

Table S1. Main features from the antimicrobial susceptibility studies included in this review that targeted the major BRD-related bacteria: *Mannheimia haemolytica, Pasteurella multocida, Histophilus somni*, and *Mycoplasma bovis*

Samples origin and sampling period	Type and no. of samples	Sampling points and study AMR goals	Bacteria targeted and no. isolates tested	ASTs protocol	Highlights of the study	Ref
			***Pasteurellaceae* AMR active surveillance studies**			
Two commercial feedlots, southern Alberta, CA. 2008–2009	NPS, general feedlot cattle population. n = 4,548	At feedlot entry/exit. AMR investigation of beef industry relevant antimicrobials.	Mh, n = 416	DD	Low Mh AMR at entry and exit.	[1] [1]
Four commercial feedlots, southern Alberta, Canada. 2007–2010	NPS, general feedlot cattle population. n = 5,814	At feedlot entry and ≥ 60 DOF. TUL resistance evaluation 12 months after its approval in Canada.	Mh, n = 4,548	AD (n = 4,548) and BM (n = 5)	Low TUL-resistance that did not change over study period.	[2]
Two commercial feedlots, southern Alberta, Canada. 2008–2009	NPS, BRD-diseased and healthy feedlot cattle. n = 90	At feedlot: healthy at entry and exit, BRD-diseased during feeding period. Mh AMR investigation associated with BRD.	Mh, healthy n = 49, sick n = 41	BM	Overall low Mh AMR; higher AMR among isolates from sick animals as per healthy.	[3] [1]
Four commercial feedlots, southern and central Alberta, Canada. 2007–2010	DNPS, general feedlot cattle population. n = 5,968	At feedlot entry and > 30 DOF. Risk factors associated with Mh AMR and cattle health outcome.	Mh, n = 2,989	DD (n = 1,789) and BM (n = 2,833)	Overall low Mh AMR.	[4]
One commercial stocker operation, central Georgia, USA. 2016	DNPS, general feedlot cattle population. n = 169	At feedlot entry and 10-14 DOF or when diagnosed with BRD (n = 3). Determine Mh AMR at entry and later in the feeding period.	Mh, n = 79, 366 [2]	DD	Overall low AMR at entry that increased substantially after feedlot placement.	[5]
One commercial feedlot, Mississippi, USA. 2016	NPS, general feedlot cattle population. n = 50	At feedlot entry and 7, 14, and 21 DOF. Determine Mh AMR and MDR at entry and during first 21 DOF.	Mh, n = 5, 27, 44, 40 [2]<br>Pm, n = 6<br>Hs, n = 0	DD (Mh and Pm) and BM (Mh)	Mh AMR increased after feedlot placement. High proportion of MDR Mh at day 7 was unexpected.	[6]
Three commercial cow-calf ranches, southern Alberta, Canada. 2016	DNPS, general beef cattle population. n = 120	At calf-ranch vaccination, feedlot entry, and 40 DOF. Determine the origin of AMR in respiratory bacteria.	Mh, n = 25, 16, 29 [2]<br>Pm, n = 1, 21, 59 [2]<br>Hs, n = 1, 0, 30 [2]	BM	AMR can increase from calf-ranch to feedlot varying across herds. Overall low Mh AMR.	[7]

Ten commercial feedlots in Alberta, Canada. 2017–2019	DNPS, general beef cattle population. n = 2,824	At feedlot processing. Investigate the epidemiology and risk factors for AMR at feedlot entry.	Mh, n = 490 Pm, n = 515 Hs, = 241	BM	Dairy-type feedlot cattle had higher AMR as per beef-type. Beef-type cattle sourced from backgrounding operations had higher AMR as per auction or ranch-direct calves.	[8]
Twenty-two commercial cow–calf operations in Alberta, Canada. 2017	DNPS, general beef cattle population. n = 1,666	At spring processing, weaning or feedlot processing, and feedlot re-processing. Identify risk factors associated with AMR including AMU.	Mh, n = 1, 30, 27 [2] Pm, n = 24, 88, 38 [2] Hs, = 0, 10, 20 [2]	BM	Metaphylactic macrolides at feedlot entry were associated with higher macrolide MICs later in the feeding period.	[9]
		***Pasteurellaceae* AMR passive surveillance studies**				
Oklahoma Animal Disease Diagnostic Laboratory, USA 1994–2002	Lung tissue or swab from bovine pneumonia	6–18-month-old beef cattle Investigate AMR of Mh, Pm, and Hs from bovine pneumonia	Mh, n = 390 Pm, n = 292 Hs, n = 160	DD	Mh and Pm susceptibilities to SPE, ERY, FFN, and TIL declined over time	[10]
Twenty-four veterinary diagnostic laboratories across USA and Canada 2000–2009	Respiratory samples from BRD clinical cases or mortalities	Track AMR trends from BRD cases in USA and CA	Mh, n = 2,977 Pm, n = 3,291 Hs, n = 1,844	BM	The TIL and TUL MIC50 and MIC90 of Mh, Pm, and Hs decreased Decrease of FFN susceptibility among Mh, Pm, and Hs	[11]
Kansas State Veterinary Diagnostic Laboratory, USA 2009–2011	Lung tissue from BRD clinical cases	Investigate Mh MDR from BRD cases	Mh, n = 389	BM	The number of isolates resistant to 5 antimicrobials increased over time	[12]
Commercial feedlots in Alberta (Canada), Texas, and Nebraska (USA)	NPS and lung tissue from BRD mortalities presenting fibrinous pneumonia n = 68	During feeding period Characterization of AMR of BRD bacteria from BRD mortalities from feedlots located in CA and USA	Mh, n = 55 Pm, n = 8 Hs, n = 10	BM	Overall high AMR among isolates of the three bacterial species Presence of ICE in Mh, Pm, and Hs from USA	[13]
Sixty commercial feedlots in southern Alberta, Canada 2014–2015	Samples from BRD clinical cases (DNPS) and mortalities (different types of samples)	During feeding period Investigation of AMR in BRD bacteria from clinical cases and mortalities	Mh, n = 251 Pm, n = 118 Hs, n = 80	BM	High AMR to macrolides (Mh, Pm) and oxytetracycline (Mh, Pm, Hs)	[14]

	n = 740					
Four commercial feedlots in southern Alberta, Canada 2015–2016	TTA from BRD clinical cases and healthy beef cattle n = 210 diseased n = 107 healthy	During feeding period Investigation of AMR in BRD bacteria from clinical cases and healthy beef cattle	Mh, n = 78 Pm, n = 135 Hs, n = 63	BM	High AMR to macrolides (Mh, Pm), oxytetracycline (Mh, Pm, Hs), and penicillin (Hs) Higher Mh and Hs AMR isolated from sick animals as per healthy	[15]
***Mycoplasma bovis* AMR active and passive surveillance studies**						
Different regions across USA 2002–2003	Four different types of tissues, including respiratory samples, from clinical cases from beef and dairy cows	Determination of the antimicrobial activity against drugs used to treat calves in USA	Mb, n = 223	BM	High MIC50 and MIC90 for macrolides [3]	[16]
One commercial feedlot in Saskatchewan, Canada 2007–2008	DNS (healthy and diseased) and lung/joint (mortalities) n = 122 healthy n = 111 morbidities and mortalities	During first 130 DOF Determine AMR from clinical cases and mortalities	Mb, n = 51	BM	High TIL MIC50	[17]
Sixty commercial feedlots in southern Alberta, Canada 2015–2016	Samples from BRD clinical cases (DNPS) and mortalities (different types of samples) n = 740	During feeding period Investigation of AMR in BRD bacteria from clinical cases and mortalities	Mb, n = 226	BM	High macrolide MIC50	[14]
Animal Health Laboratory at the University of Guelph, Canada 1978–2019	Samples from clinical cases; mainly respiratory (62%), milk, and joint samples	Explore MIC changes over time	Mb, n = 210	BM	MIC50 increase for TETs, TIL, and TYL [4]	[18]
Western Canada and Idaho, USA 2006–2018	DNPS (healthy and diseased*), lung, and joint *Mb pneumonia or CPPS	During feeding period Explore antimicrobial susceptibility from healthy, diseased, and dead cattle	Mb, n = 211	BM	High macrolide MICs even among healthy cattle MICs tended to increase over time and were higher among death cattle as per healthy	[19]

Ten commercial feedlots in Alberta, Canada. 2017–2019	DNPS, general beef cattle population. n = 2,824	At feedlot processing. Investigate the epidemiology and risk factors for AMR at feedlot entry.	Mb, n = 222	BM	High macrolide MICs regardless of cattle type.	[8]
Twenty-two commercial cow–calf operations in Alberta, Canada. 2017	DNPS, general beef cattle population. n = 1,666	At spring processing, weaning or feedlot processing, and feedlot re-processing. Identify risk factors associated with AMR including AMU.	Mb, n = 2, 19, 28 [2]	BM	High macrolide MICs regardless of sampling point. Increase of FFN-resistance during the feeding period.	[9]

AD, agar dilution; AMR, antimicrobial resistance; AMU, antimicrobial use; AST, antimicrobial susceptibility test; BM, broth microdilution; BRD, bovine respiratory disease; CPPS, chronic pneumonia and polyarthritis syndrome; DD, disk diffusion; DNPS, deep nasopharyngeal swabs; DNS, deep nasal swab; DOF, days on feed; ERY, erythromycin; FFN, florfenicol; H, healthy; Hs, *Histophilus somni*; ICE, integrative and conjugative element; LRT, lower respiratory tract; Mb, *Mycoplasma bovis*; MDR, multidrug resistance; Mh, *Mannheimia haemolytica*; MIC, minimum inhibitory concentration; no., number; NPS, nasopharyngeal swabs; Pm, *Pasteurella multocida*; SPE, spectinomycin; TETs, tetracyclines; TIL, tilmicosin; Tp, Trueperella pyogenes; TTA, trans-tracheal aspiration; TUL, tulathromycin; tx, treatment; TYL, tylosin; URT, upper respiratory tract.

[1] Klima et al. (2014a) characterized a subset of isolates already tested on Klima et al. (2011).

[2] Number of bacterial isolates tested by sampling point.

[3] The MIC distributions obtained from isolates recovered from different types of tissues showed remarkable similarity.

[4] The MIC distributions obtained from isolates recovered from different breeds or tissues did not differ.

[1] Klima et al. 2011; [2] Alexander et al. 2013; [3] Klima et al. 2014a; [4] Noyes et al. 2015; [5] Snyder at al. 2017; [6] Woolums et al. 2018; [7] Guo et al. 2020; [8] Andres-Lasheras et al. 2021; [9] Nobrega et al., 2021; [10] Welsh et al. 2004; [11] Portis et al. 2012; [12] Lubbers and Hanzlicek. 2013; [13] Klima et al. 2014b; [14] Anholt et al. 2017; [15] Timsit et al. 2017; [16] Rosenbusch et al. 2005; [17] Hendrick et al. 2013; [18] Cai et al. 2019; [19] Jelinski et al. 2020.
